# Structural transition of parenthood among Chinese nulliparous couples with planned pregnancies, 2013–2019

**DOI:** 10.1186/s12889-023-17380-2

**Published:** 2023-12-04

**Authors:** Long Wang, Chunying Han, Xinyi Lv, Shuai Zeng, Rongwei Mu, Yuzhi Deng, Wenlu Xie, Jiaxin Huang, Siyu Wu, Ya Zhang, Hongguang Zhang, Yuan He, Zuoqi Peng, Yuanyuan Wang, Haiping Shen, Qiaomei Wang, Yiping Zhang, Donghai Yan, Ying Yang, Xu Ma

**Affiliations:** 1 Institute of Epidemiology and Statistics, School of Public Health, Lanzhou University, Lanzhou, China; 2 National Research Institute for Family Planning, Beijing, China; 3 National Human Genetic Resources Centre, Beijing, China; 4 Graduate School of Peking Union Medical College, Beijing, China; 5 Department of Maternal and Child Health, National Health Commission of the PRC, Beijing, China

**Keywords:** Parenthood postponement, Within-population heterogeneity, China

## Abstract

**Background:**

The postponement of parenthood is a global public health issue that has received attention of many public health experts. However, few studies have investigated the postponement in marriage age, marriage and conception interval, and pregnancy age in terms of demographic and regional heterogenicities.

**Methods:**

This is a cross-sectional, registry-based study, and a total of 13 894 601 nulliparous couples who participated in the National Free Pre-Pregnancy Check-ups Project and became pregnant during 2013–2019 were included. We calculated annual percentage change and forest plots for marriage age, marriage and conception interval, and pregnancy age.

**Results:**

Late marriage (marriage age ≥ 35 years), long marriage and conception interval (marriage and conception interval ≥ 2 years), and advanced pregnancy (pregnancy age ≥ 35 years) increased from 1.20%, 22.01%, and 1.88% in 2013 to 1.69%, 32.75%, and 2.79% in 2019, respectively. The corresponding annual percentage changes were 6.55%, 8.44%, and 8.17%. Participants without higher education had a higher annual percentage change, but comparable prevalence for long marriage and conception interval with participants with higher education. Participants residing in second- or new first-tier cities, and the northeast of China who had a higher prevalence of parenthood postponement also had higher corresponding annual percentage changes.

**Conclusions:**

Structural postponement of parenthood with demographic and regional heterogenicities was observed among Chinese nulliparous couples with planned pregnancies during 2013–2019. Inclusive and comprehensive parenting support should be developed and implemented in mainland China to minimize the negative health effects arising from the postponement, especially for couples without higher education and living in new first/second-tier cities or the northeast China.

**Supplementary Information:**

The online version contains supplementary material available at 10.1186/s12889-023-17380-2.

## Background

The postponement of parenthood is a global public health concern, affecting population structure, and is a consequence of increased female emancipation. The use of contraceptive technology since the early 1960s has reshaped human fertility and enabled females to take charge of their reproductive lives. Delayed parenthood provides women of reproductive age with ample time to pursue higher education, establish career, enjoy couple time, and enhance economic status. Research indicates that labor force participation, ideational or value shifts, and gender equity, partnerships, housing and economic uncertainty are involved in the postponement. Furthermore, additional sociocultural determinants contribute to the postponement. [1] Moreover, birth control has also effectively contributed to this phenomenon, especially in mainland China, along with the second demographic transition driven mainly by ideational shifts [2, 3].

The postponement could pose a risk to the health of pregnancy in humans. The delaying of age at first birth, both by mothers and fathers, carries an increased risk of infertility, multiple pregnancy complications, and poor fetal and neonatal outcomes [4–6]. The postponement has persisted for several decades with variations across different countries. Late childbearing was a common occurrence until the 1950s, and it continued throughout the twentieth century in developed countries [7]. In the United States(1970–2006), the age of first-time mothers has increased by 0.100 years annually, while in Denmark (1987–2018), it has increased by 0.110 years annually. Similarly, in China (2000–2010), Japan (1990–2015), and the United Kingdom (2010–2016), the annual increased has been recorded as 0.19 years, 0.148 years, and 0.167 years, respectively [8–12]. The delayed age of first marriage has been widely reported as the primary cause of postponement [13, 14]. Compared to early marriage societies, postponement of marriage could potentially push new couples to shorten the interval between marriage and first birth to compensate for the delay [15]. This, in turn, could result in underprepared planned pregnancy, leading to an increased risk in the coming pregnancy. While assisted reproductive technologies can compensate for infertility caused by postponement, many individuals are unaware of the potential health consequences [1].

Numerous countries and regions face the challenge of an aging population and declined fertility rates, highlighting the need to reduce the postponement of parenthood. The identified factors suggest that social policies play a key role in effectively addressing the issue. Despite the implementation of various social policies, such as direct cash payments, indirect transfers, and improved work-family compatibility, the postponement of parenthood persists. This suggests a need to empower individuals toward an organized and planned parenthood, and creates a balanced work-family condition at the micro and family level. Since multiple populations have been evaluated for postponement disparity, the present study endeavors to examine the intrapopulation diversity of the transition (including levels and annual changes) across demographic, socioeconomic, and spatial characteristics.

Mainland China, the study area, has a sizeable reduction in birth rates since the early 1970s. Both the country’s one-child family planning policy and sociocultural transition in the country were significant contributors to the population decline. In response, authorities implemented a two-child policy in 2015 and a three-child policy in 2021. Measures were taken to address the negative effects, but unfortunately, 2022 saw a further decrease in population growth. Due to the lack of significant changes in financial, social, and cultural factors, it is anticipated that the birth rate will remain low [16]. This transition of parenthood in mainland China is predicted to continue in the coming years. Consequently, this delay would decrease the total fertility rate and produce a reshaped population structure.

In this study, we proposed to characterize the transition of marriage age, MCI, and pregnancy age across demographic, socioeconomic, and spatial characteristics among Chinese nulliparous couples with planned pregnancies. Our investigate will reveal the with-in population heterogeneity of this structural transition. The heterogeneity could be used to explore potential sociocultural drivers of transition and supply policy implications accordingly.

## Methods

### Study design and population

This cross-sectional, registry-based study used data obtained from the National Free Pre-Pregnancy Check-ups Project (NFPCP). The NFPCP is a free and ongoing national preconception health examination and counseling service for couples planning to become pregnant within 6 months since 2010. Couples were recruited by local community officers. Eligible couples planning to conceive in the next 6 months will visit local maternal and child care service centers for preconception health examination. The project was conducted only in pilot areas before 2013, and then expanded to all counties in mainland China afterward. The detailed design, organization and implementation of the NFPCP have been described elsewhere [17].

Briefly, baseline information for the preconception health examination, including age, age at marriage, education, home address, and ethnicity of the couples, was collected by uniformly trained health workers using a structured questionnaire with face-to-face interviews at local maternal and child service centers. After the completion of the examination, two rounds of follow-up via telephone were conducted by trained health workers. Only information from the first round of follow-up was used in the current study, which was conducted to determine conception status every 3 months within 1 year of the baseline examination until the conception status was confirmed. Women who became pregnant were asked to return to the healthcare center 2 months after their last menstrual period (LMP) for an undergo ultrasound scan to confirm the pregnancy.

Nulliparous female participants who became pregnant between 1 January 2013 and 31 December 2019, and their husbands were included in the current study. After selecting participants with LMP aged 20–49 years and excluding Tibetan participants, 13 894 601 participants were included in the primary analysis. Nulliparous couples in this study were defined as women of reproductive age who had never given birth to a live child and their husbands.

### Outcomes and baseline characteristics

The outcomes used in the current study include age at marriage, MCI, and age at pregnancy in both quantitative and qualitative forms. MCI is defined by the difference between marriage time and LMP in years. Participants with marriage age and pregnancy age ≥ 35 years were defined as late marriage and advanced pregnancy, respectively, and participants with MCI ≥ 2 years were defined as long MCI. As participants with spontaneous abortion, therapeutic induced labor, or stillbirth have no date of delivery, we used LMP as the reference point for defining MCI.

Baseline characteristics included sex (male, female), higher education (yes, no), household registration type (urban, rural), Han ethnicity (yes, no), body mass index (BMI), occupation (worker, farmer, others), provincial gross domestic product (GDP) per capita in 2016 (< 40 000, 40 000-, 50 000-, and 70 000- CNY; source: data.stats.gov.cn), tier of cities (defined according to the 2020 ranking of cities for commercial attractiveness, including first-tier cities, new first-tier cities, second-tier cities, third-tier cities, fourth-tier cities, and fifth-tier cities, see supplementary materials for more details), seven regions defined by province location are northeast (Liaoning, Jilin, and Heilongjiang), north (Beijing, Tianjin, Hebei, Shanxi, and Inner Mongolia), northwest (Shaanxi, Gansu, Qinghai, Ningxia, and Xinjiang), central (Henan, Hubei, and Hunan), east (Shanghai, Jiangsu, Zhejiang, Anhui, Fujian, Jiangxi, and Shandong), south (Guangdong, Guangxi, and Hainan), and southeast (Chongqing, Sichuan, Guizhou, and Yunnan), and provinces. BMI was classified into underweight (< 18.5 kg/m^2^), normal weight (18.5 kg/m^2^ ≤ to < 24.0 kg/m^2^), overweight (24.0 kg/m^2^ ≤ to < 28.0 kg/m^2^) and obesity (≥ 28.0 kg/m^2^).

### Statistical analysis

We used numbers (N) and percentages (%) to describe the baseline characteristics of the participants. Differences in baseline characteristics across LMP years were tested by the chi-square test.

The average levels with the corresponding confidence intervals (95% CI) of age at marriage age, MCI, and age at pregnancy in husbands and wives were calculated. The prevalence with 95% CI of late marriage, long MCI, and advanced pregnancy in husbands and wives was calculated.

We used Likert plots to investigate the transition of age at marriage and at age pregnancy age by years, and a cumulative bar chart for MCI. We used annual change (AC) with 95% CI to measure the temporal trends of marriage age, MCI, and pregnancy age during 2013–2019. The AC was obtained by fitting a simple linear model on average levels. We used APC with 95% CI to measure temporal trends of late marriage, long MCI, and advanced pregnancy during 2013–2019. The APC was obtained by fitting a simple linear model to the logarithm o the rates. We also calculated changes in average levels and rates between 2013 and 2019. We used forest plots to explore within-population heterogeneity in marriage age, MCI, and pregnancy age transitioning.

Statistical analyses were performed with R 4.0.2 (https://www.r-project.org). Two-sided *P* values of < 0.05 were considered statistically significant. Geographical mapping was drawn with ArcGIS 10.2.

## Results

The detailed sample size and demographic characteristics are shown in Table 1. From 2010 to 2019, participants were more likely to be highly educated, have urban residency, and be a minority, and less likely to be farmer.

**Table 1:** Descriptive statistics of the included participants

Characteristic	2013	2014	2015	2016	2017	2018	2019	P
**N**	2 984 537	2 702 271	2 543 752	1 986 583	1 578 652	1 342 182	756 624	
**Gender**								0.0306
Male	1 493 737 (50.05)	1 353 948 (50.10)	1 275 096 (50.13)	995 527 (50.11)	791 009 (50.11)	673 573 (50.18)	380 231 (50.25)	
Female	1 490 800 (49.95)	1 348 323 (49.90)	1 268 656 (49.87)	991 056 (49.89)	787 643 (49.89)	668 609 (49.82)	376 393 (49.75)	
**Higher Education**								< 0.001
Yes	1 227 629 (41.13)	1 141 749 (42.25)	1 183 161 (46.51)	996 669 (50.17)	824 397 (52.22)	749 145 (55.82)	458 386 (60.58)	
No	1 667 334 (55.87)	1 474 310 (54.56)	1 257 520 (49.44)	894 858 (45.05)	661 442 (41.90)	441 363 (32.88)	221 961 (29.34)	
NA	89 574 (3.00)	86 212 (3.19)	103 071 (4.05)	95 056 (4.78)	92 813 (5.88)	151 674 (11.30)	76 277 (10.08)	
**Registering Type**								< 0.001
Urban	385 769 (12.93)	364 856 (13.50)	420 610 (16.54)	364 720 (18.36)	316 787 (20.07)	285 679 (21.28)	186 253 (24.62)	
Rural	2 598 768 (87.07)	2 337 415 (86.50)	2 123 142 (83.46)	1 621 863 (81.64)	1 261 865 (79.93)	1 056 503 (78.72)	570 371 (75.38)	
**Han ethnicity**								< 0.001
Yes	2 782 714 (93.24)	2 508 625 (92.83)	2 357 085 (92.66)	1 829 190 (92.08)	1 450 324 (91.87)	1 215 658 (90.57)	680 759 (89.97)	
No	161 299 (5.40)	159 076 (5.89)	149 790 (5.86)	128 932 (6.49)	101 089 (6.40)	74 443 (5.55)	54 494 (7.21)	
NA	40 524 (1.36)	34 570 (1.28)	36 877 (1.45)	28 461 (1.43)	27 239 (1.73)	52 081 (3.88)	21 371 (2.82)	
**Occupation**								< 0.001
Worker	31 8505 (10.67)	290 360 (10.75)	279 245 (10.98)	223 949 (11.27)	193 072 (12.23)	162 226 (12.09)	96 265 (12.72)	
Farmer	1 987 853 (66.61)	1 778 461 (65.81)	1 549 810 (60.93)	1 108 120 (55.78)	796 622 (50.46)	554 898 (41.34)	262 843 (34.74)	
Others	576 527 (19.32)	533 179 (19.73)	595 738 (23.42)	542 173 (27.29)	479 446 (30.37)	448 328 (33.40)	309 218 (40.87)	
NA	101 652 (3.41)	100 271 (3.71)	118 959 (4.68)	112 341 (5.65)	109 512 (6.94)	176 730 (13.17)	88 298 (11.67)	
**BMI, kg/m** ^**2**^								< 0.001
Underweight	325 468 (10.91)	293 984 (10.88)	272 783 (10.72)	210 711 (10.61)	166 835 (10.57)	139 327 (10.38)	78 498 (10.37)	
Normal weight	2 058 868 (68.98)	1 856 727 (68.71)	1 717 730 (67.53)	1 318 084 (66.35)	1 020 808 (64.66)	858 672 (63.98)	472 182 (62.41)	
Overweight	458 612 (15.37)	425 900 (15.76)	427 310 (16.80)	351 057 (17.67)	294 957 (18.68)	259 175 (19.31)	151 874 (20.07)	
Obesity	99 640 (3.34)	92 890 (3.44)	99 454 (3.91)	84 305 (4.24)	76 315 (4.83)	71 773 (5.35)	45 800 (6.05)	
NA	41 949 (1.41)	32 770 (1.21)	26 475 (1.04)	22 426 (1.13)	19 737 (1.25)	13 235 (0.99)	8 270 (1.09)	
**GDP per Capita, CNY**								< 0.001
<40 000	588 070 (19.70)	591 378 (21.88)	571 745 (22.48)	422 681 (21.28)	370 314 (23.46)	327 807 (24.42)	204 837 (27.07)	
40 000-	1 215 980 (40.74)	1 131 389 (41.87)	1 054 060 (41.44)	788 107 (39.67)	476 105 (30.16)	366 112 (27.28)	124 215 (16.42)	
50 000-	510 753 (17.11)	355 903 (13.17)	320 604 (12.60)	253 072 (12.74)	254 413 (16.12)	256 028 (19.08)	170 679 (22.56)	
70 000-	669 734 (22.44)	623 601 (23.08)	597 343 (23.48)	522 723 (26.31)	477 820 (30.27)	392 235 (29.22)	256 893 (33.95)	
**Tier of Cities**								< 0.001
First	75 261 (2.52)	59 833 (2.21)	69 254 (2.72)	68 729 (3.46)	65 722 (4.16)	72 985 (5.44)	57 632 (7.62)	
New First	181 245 (6.07)	148 734 (5.50)	162 498 (6.39)	134 827 (6.79)	126 622 (8.02)	106 594 (7.94)	75 777 (10.02)	
Second	277 791 (9.31)	244 672 (9.05)	243 446 (9.57)	191 282 (9.63)	177 413 (11.24)	118 360 (8.82)	96 928 (12.81)	
Third	1 148 041 (38.47)	1 057 004 (39.12)	990 884 (38.95)	751 313 (37.82)	594 469 (37.66)	523 529 (39.01)	239 561 (31.66)	
Fourth	866 655 (29.04)	790 412 (29.25)	700 886 (27.55)	526 160 (26.49)	345 621 (21.89)	291 887 (21.75)	150 080 (19.84)	
Fifth	435 544 (14.59)	401 616 (14.86)	376 784 (14.81)	314 272 (15.82)	268 805 (17.03)	228 827 (17.05)	136 646 (18.06)	
**Region**								< 0.001
Northeast	43 591 (1.46)	30 136 (1.12)	32 337 (1.27)	30 367 (1.53)	30 878 (1.96)	32 920 (2.45)	20 337 (2.69)	
North	245 107 (8.21)	194 526 (7.20)	187 046 (7.35)	133 008 (6.70)	113 058 (7.16)	39 817 (2.97)	56 865 (7.52)	
Northwest	166 687 (5.59)	158 257 (5.86)	160 391 (6.31)	143 326 (7.21)	139 111 (8.81)	153 082 (11.41)	124 072 (16.40)	
Central	1 092 245 (36.60)	1 040 056 (38.49)	963 398 (37.87)	726 295 (36.56)	447 145 (28.32)	381 218 (28.40)	74 501 (9.85)	
East	636 522 (21.33)	519 230 (19.21)	520 971 (20.48)	372 843 (18.77)	358 272 (22.69)	330 706 (24.64)	261 402 (34.55)	
South	519 496 (17.41)	499 717 (18.49)	446 844 (17.57)	392 466 (19.76)	340 973 (21.60)	283 624 (21.13)	169 465 (22.40)	
Southwest	280 889 (9.41)	260 349 (9.63)	232 765 (9.15)	188 278 (9.48)	149 215 (9.45)	120 815 (9.00)	49 982 (6.61)	

*Note*: BMI = body mass index; GDP = gross domestic product; NA = not available. Data are presented as N (%)

**Fig. 1:**
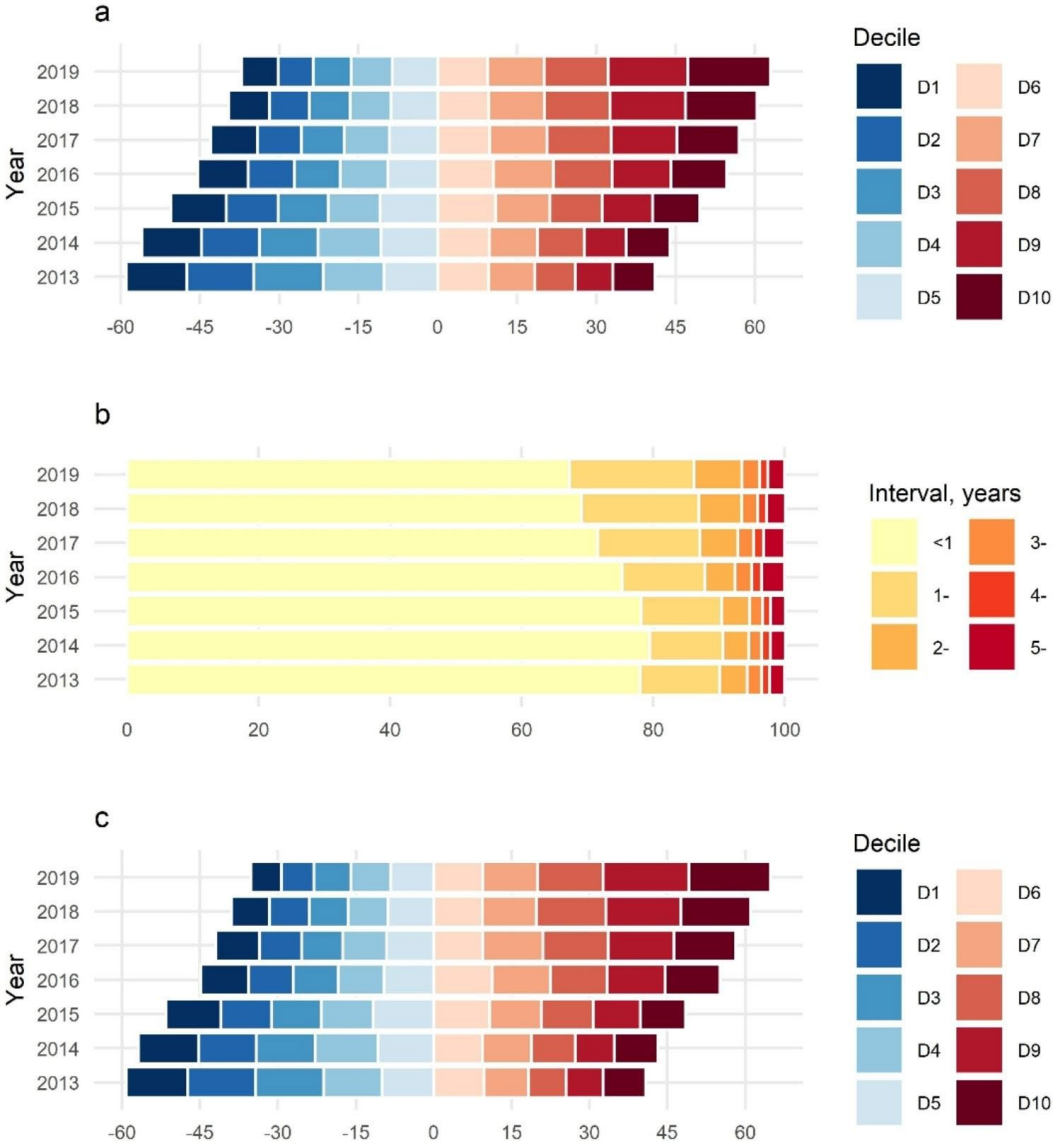
Percentage shift of marriage age, marriage and conception interval, and pregnancy age among participants. **a** = Marriage Age; **b** = Marriage and Conception Interval; **c** = Pregnancy Age. Deciles of marriage age in years are 21.63, 22.60, 23.41, 24.21, 24.98, 25.72, 26.58, 27.65, and 29.40. Deciles of pregnancy age in years are 22.08, 23.12, 23.98, 24.82, 25.61, 26.43, 27.34, 28.49, and 30.42

The summary graphs in Fig. 1 show that marriage age (Fig. 1A) and pregnancy age (Fig. 1C) were transitioning into postponed parenthood with an increasing percentage of longer MCI (Fig. 1B).

Of the included participants, the mean marriage age, MCI, and pregnancy age were 25.29 (95% CI: 25.29–25.29), 0.90 (0.90–0.90), and 26.03 (26.03–26.04) years, respectively. The percentages of late marriage, long MCI, and advanced pregnancy were 1.31% (1.31-1.32%), 24.29% (24.26-24.31%), and 2.17% (2.16-2.17%), respectively (Table 2).

**Table 2:** Trends of marriage age, marriage and conception interval, and pregnancy age among participants

Year	Mean (95% CI)	Prevalence in % (95% CI)
**Marriage Age**		
Total	25.29 (25.29–25.29)	1.31 (1.31–1.32)
2013	24.78 (24.78–24.79)	1.20 (1.18–1.21)
2014	24.94 (24.94–24.95)	1.19 (1.18–1.21)
2015	25.20 (25.19–25.20)	1.19 (1.17–1.20)
2016	25.50 (25.50-25.51)	1.39 (1.37–1.41)
2017	25.69 (25.68–25.69)	1.45 (1.42–1.47)
2018	25.96 (25.96–25.97)	1.57 (1.54–1.59)
2019	26.20 (26.19–26.21)	1.69 (1.66–1.73)
*P*_*trend*_	< 0.001	< 0.001
AC	0.24 (0.23–0.26)	-
APC	-	6.55 (4.79–8.35)
**Marriage and Conception Interval**		
Total	0.90 (0.90–0.90)	24.29 (24.26–24.31)
2013	0.84 (0.84–0.84)	22.01 (21.96–22.07)
2014	0.80 (0.80–0.80)	20.59 (20.53–20.65)
2015	0.83 (0.82–0.83)	21.82 (21.76–21.88)
2016	0.99 (0.98–0.99)	24.73 (24.66–24.80)
2017	1.03 (1.03–1.03)	28.46 (28.37–28.54)
2018	1.03 (1.03–1.03)	30.88 (30.79–30.97)
2019	1.05 (1.05–1.06)	32.75 (32.63–32.87)
*P*_*trend*_	> 0.001	> 0.001
AC	0.05 (0.03–0.06)	-
APC	-	8.44 (5.92–11.02)
**Pregnancy Age**		
Total	26.03 (26.03–26.04)	2.17 (2.16–2.17)
2013	25.46 (25.46–25.47)	1.88 (1.87–1.90)
2014	25.61 (25.61–25.62)	1.88 (1.87–1.90)
2015	25.88 (25.88–25.89)	1.87 (1.85–1.89)
2016	26.30 (26.30-26.31)	2.44 (2.42–2.46)
2017	26.55 (26.54–26.55)	2.59 (2.56–2.61)
2018	26.80 (26.80-26.81)	2.66 (2.64–2.69)
2019	27.14 (27.14–27.15)	2.79 (2.75–2.83)
*P*_*trend*_	< 0.001	> 0.001
AC	0.29 (0.27–0.31)	-
APC	-	8.17 (5.34–11.07)

*Note*: AC = annual change; APC = annual percent change

In detail, mean marriage age increased from 24.78 years in 2013 to 26.20 years in 2019 (*P*_trend_ < 0.001), reaching 0.24 (0.23–0.26) years annually; and late marriage increased from 1.20% in 2013 to 1.69% in 2019 (*P*_trend_ <0.001) by 6.55% (4.79-8.35%) per year. The MCI increased from 0.84 years in 2013 to 1.05 years in 2019 (*P*_trend_ >0.001) by 0.05 (0.03–0.06) years annually; and the long MCI increased from 22.01% in 2013 to 32.75% in 2019 (*P*_trend_ >0.001) by 8.44% (5.92-11.02%) per year. Pregnancy age increased from 25.46 years in 2013 to 27.14 years in 2019 (*P*_trend_ < 0.001) by 0.29 (0.27–0.31) years annually; and advanced pregnancy age increased from 1.88% in 2013 to 2.79% in 2019 (*P*_trend_ >0.001) by 8.17% (5.34-11.07%) per year. Even in participants without a history of pregnancy, the results presented identical trends. (see supplementary Table S1)

**Fig. 2:**
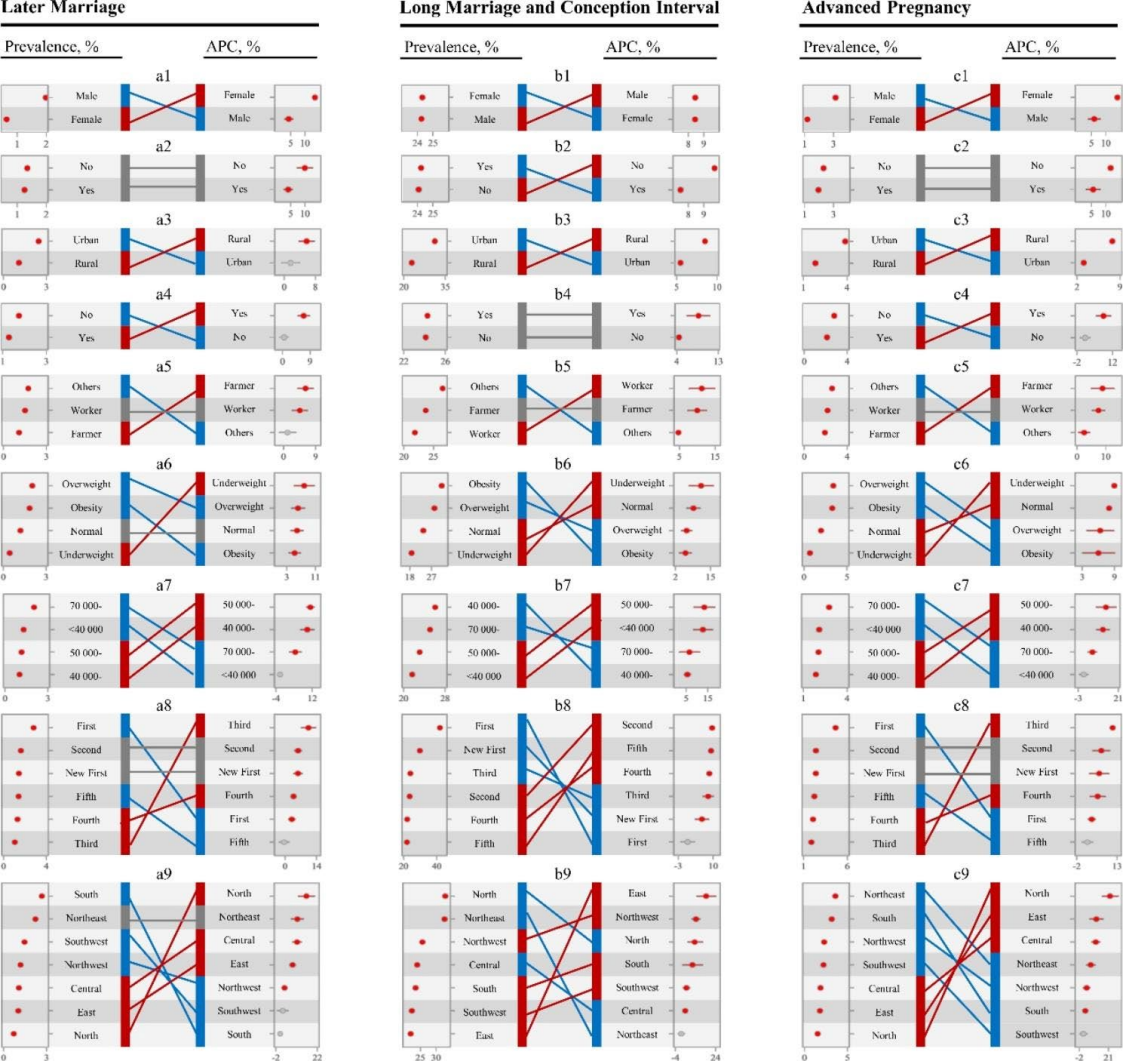
Population heterogeneity of late marriage, long marriage and conception interval, and advanced pregnancy among participants. **a** = Late Marriage; **b** = Long Marriage and Conception Interval; **c** = Advanced Pregnancy; 1 = Gender; 2 = Higher Education; 3 = Registering Type; 4 = Han ethnicity; 5 = Occupation; 6 = BMI; 7 = GDP per capita; 8 = City of cities; 9 = Region. For each panel, the prevalence during 2013–2019 is presented on the left side, and the corresponding annual percent change is presented on the right side

**Fig. 3:**
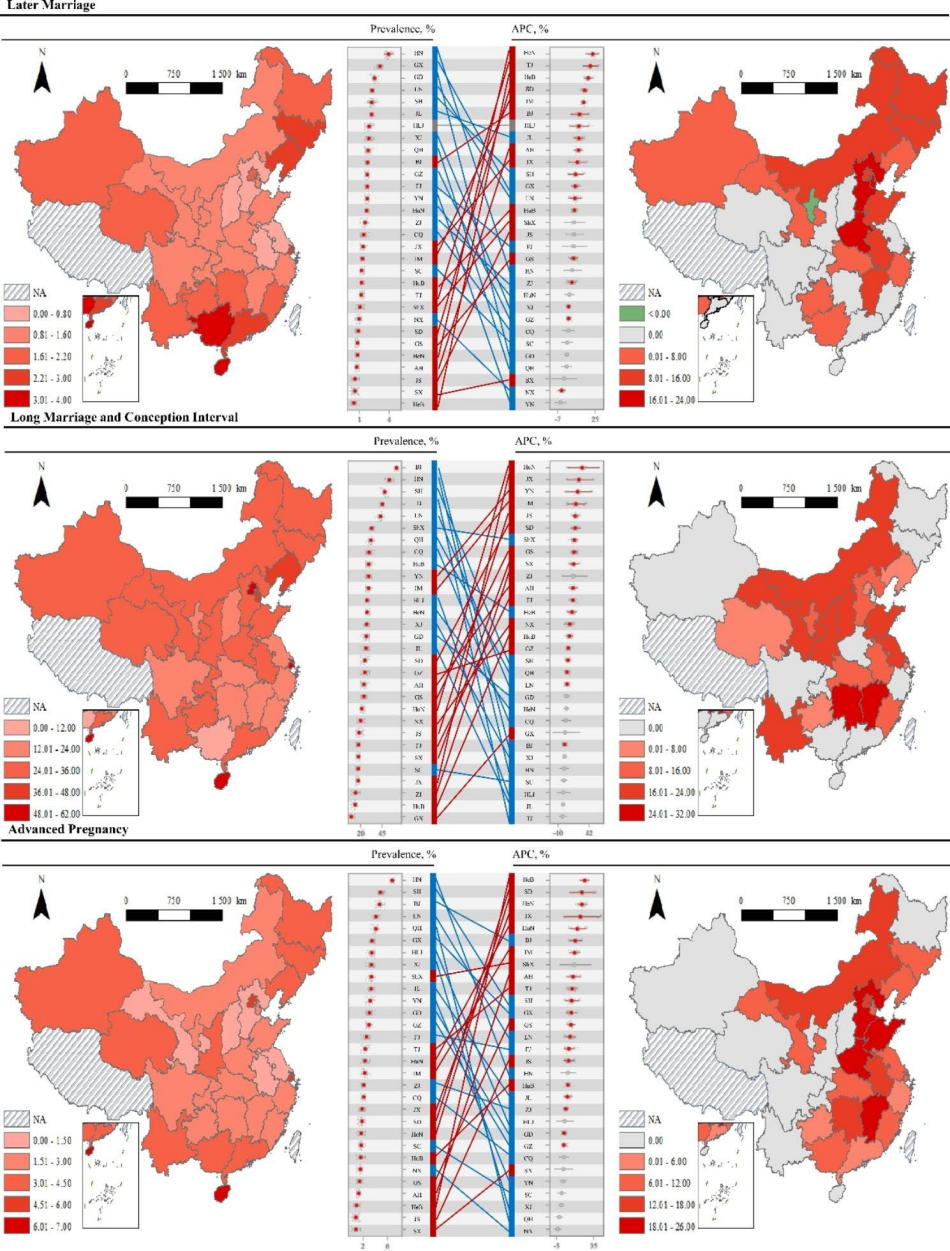
Provincial heterogeneities of late marriage, long marriage and conception interval, and advanced pregnancy among participants. Provinces with a ***P***_*trend*_ <0.05 in figures of annual percent change are filled with gray. Short names of provinces are used here, where AH is Anhui, BJ is Beijing, CQ is Chongqing, FJ is Fujian, GD for Guangdong, GS is Gansu, GX is Guangxi, GZ is Guizhou, HN is Hainan, HeB is Hebei, HeN is Henan, is HLJ for Heilongjiang, HuB is Hubei, HuN is Hunan, IM is Inner Mongolia, JL is Jilin, JS is Jiangsu, JX is Jiangxi, LN is Liaoning, NX is Ningxia, QH ir Qinghai, SC is Sichuan, SD is Shandong, SH is Shanghai, ShX is Shaanxi, SX is Shanxi, TJ is Tianjin, XJ for Xinjiang, YN for Yunnan, and ZJ for Zhejiang

With-in population heterogeneities of marriage age, MCI, and pregnancy age transitions are shown in Figs. 2 and 3. Participants in subgroups with higher percentages of late marriage, long MCI, and advanced pregnancy have lower APCs, and vice versa. Although participants without higher education have a similar percentage of long MCI with participants with higher education, they have higher APC of long MCI. Participants living in second- or new first-tier cities have higher percentages and APCs of late marriage and advanced pregnancy. Participants in northeast China had a higher percentage and APC of late marriage, and participants living in northern and northwestern China have a higher percentage and growth of long MCI. No statistically significant postponement of first marriage was observed in participants of minority, urban residency, and living in fifth-tier cities, provinces with GDP per capita of < 40 000 CNY, or in southern and southeastern China. Similar results were found in participants living in first-tier cities and northeastern China for long MCI transitioning, and participants of minority and living in fifth-tier cities, southeastern China and provinces with GDP per capita of < 40,000 CNY for advanced pregnancy postponement.

Provincial heterogeneities of late marriage age, long MCI, and advanced pregnancy transitioning are presented in Fig. 3. Generally, provinces with higher prevalence of late marriage, long MCI, and advanced pregnancy during 2013–2019 were more likely to have lower APCs, and vice versa. We also noticed higher-ranked late marriage in percentages and APCs in Jilin, Heilongjiang, and Beijing. For long MCI, higher percentages and APCs coexisted in Shaanxi, Yunnan, and Inner Mongolia. For advanced pregnancy, higher percentages and APCs coexisted in Beijing, Shanghai, and Guangxi.

## Discussion

The study reveals a delayed marriage age and pregnancy age with prolonged MCI among Chinese women planned and nulliparous pregnancies during 2013–2019. Public health practitioners and population policy makers must focus attention on reproductive-aged couples with low socioeconomic status, residing in new first-tier and second-tier cities and in northeastern and northwestern China. Our results highlighted the building inclusive marriage and family planning policies to mitigate the heavy burden from socioeconomic environments and reduce the likelihood of advanced pregnancy complications for both mothers and fetuses.

Postponing parenthood has become a widespread phenomenon globally and is expected to persist for several decades. Our study conducted on Chinese couples who were planning to conceive or had never given birth revealed that delaying childbearing was marked by a rise in the age of marriage and MCI during 2013–2019. The rise in pregnancy age during 2013–2019 was about 1.53 times higher than that during 2000–2010. Additionally, it was 2.9 times higher than the increase observed in OECD counties during 1970–2008 [1, 12]. Our study indicated an even more severe situation of postponing parenthood in mainland China, with social drivers having an unrelenting and growing impacts on the parenthood transition. Late childbearing in mainland China is expected to continue increasing along with that of most developed countries [18]. The increased delay in pregnancy age is having a negative impact on both the total fertility rate and the aging population [19]. Furthermore, MCI was expected to be decreased but eventually increased. This suggests that delaying marriage may only partially contributes to later pregnancies. In addition to biological factors such as decreasing fertility rates, it is crucial to offer social assistance to newlywed couples. This may include long parental leave, equal access to high-quality nurseries, and compulsory elementary education services, and expanded and affordable health insurance coverage [20, 21]. Post-birth social support on pursuing higher education and career progression for mothers and families should be introduced. The study, conducted in mainland China, was a population-based design focusing on planned and nulliparous pregnancies. And local maternal and child service centers were requested to enroll at least the number of liver births in the previous year to participate NFPCP. The results obtained possess low bias and could be generalized to the general population. Newlyweds who planned to become pregnant had higher motivation to prepare for pregnancy and become pregnant at an appropriate age.

Planned and nulliparous pregnancies in mainland China have undergone a progressive delay in parenthood across almost all subgroups. Our study discovered that those in subgroups having higher proportions of late marriage, long MCI, or advanced pregnancy were more likely to have lower APCs, and vice versa. This finding agreed with the statistical phenomenon named regression to the mean [22]. Additionally, there were within-population disparities during the transition.

Subgroups that do not follow the rule are worthy of intensive research into the underlying reasons and of great public health concern. The current study found similar postponement of parenthood between participants with and without higher education in 2019, including later marriage, longer MCI, and advanced pregnancy. Higher education is a well-documented driver of age at first birth [1]. Given that China is one of several countries with very low out-of-wedlock birth rates, our study suggests that there are other important reasons for postponing marriage [23]. The unbearable burden of betrothal gifts may be one of the factors contributing to the postponement [24]. Although both unmarried men with and without higher education face pressure from the gifts, participants without higher education appear to face a greater burden from the gifts. Whether youth unemployment for the less educated in China contributes to deferral has not yet been well studied. Unfortunately, there was a rapid increase in late marriage and postponement of parenthood among participants with no tertiary education. Participants without higher education also had a greater increase in longer MCI, suggesting a susceptible status to postponement of parenthood, which may be associated with increased socioeconomic pressures caused by economic growth and the pursuit of higher socioeconomic standing. The longer MCI among educated participants was consistent with that in northwestern Ethiopia [25]. Educated participants had light parenthood postponement, indicating a greater ability to adapt to socioeconomic impacts on parenthood. The heterogeneity in higher education calls for strong social and policy support for newlyweds without high education or low socioeconomic status in families formation and parenthood [26]. Serious parenthood postponement was found among newlyweds in new first- and second-tier cities, which may be partly due to the booming economy and ambition of seeking higher socioeconomic status and targeted social support, such as rebuilding confidence in returning to work and continuing careers for new mothers and fathers [27, 28]. And in northeastern China, where population out-migration is a prevalent social problem, population policy makers should focus on the accelerated late marriage transition, and identify potential causes and stabilize the population [29]. The structural transition also featured as a long MCI in eastern, northern, and northwestern China, which implied parenting hesitancy and a higher burden of reproduction cost [20, 30, 31]. Additionally, precise population policies should be developed given the provincial variability of parenthood postponement in this study.

This structural transition of parenthood postponement in planned and nulliparous pregnancies of mainland China during 2013–2019 had significant implications for family planning and public health. China has the world’s fastest aging population and long-standing low fertility rate; the transition would put more pressure on the population structure and the insufficient labor force. Although the authorities have introduced a two-child policy, the number of births had fallen from 17.9 million in 2015 to 9.56 million in 2022. The postponement of parenthood can be a serious obstacle to birth. With the increased percentages of delayed marriage, longer marriage and conception interval, and advanced pregnancy, reproductive women would have decreased fertility when trying to conceive for their second child. And this decline would subsequently lengthen the inter-pregnancy intervals and limit the family size in mainland China. Our study also suggests that the number of births could not be substantially increased if couples were allowed to have four or more children in further family planning practices. We call for inclusive and comprehensive parental support policies to be developed and implemented in mainland China to mitigate the postponement of childbearing. In order to release the fertility capacity, efforts should be directed at rebuilding perceptions of parenthood, raising the awareness of timely pregnancy and the drawbacks of assistive reproductive technologies, and building a friendly and institutionally fertility-supportive social environment.

### Limitations and strengths

A particular strength of this study is that no relevant research has been conducted in this area in the past decade, and its findings have important practical implications for the development of marriage and pregnancy policies in China. Second, the heterogeneity of the follow-up population was described, in which the changes in the base values and growth rates of marriage age, MCI, and pregnancy age were described. Third, the study data were obtained from the NFPCP, which was a reliable source, and the large amount of data in this study had a high extrapolation power.

Our study had several limitations. First, this study could not analyze the specific reasons for the delayed age at marriage, MCI and age at pregnancy, and more studies are expected to investigate this issue in depth in the future. Second, our study only covered the period 2013–2019, which limit our ability to assess the long-term trends.Finally, participants in this study were predominantly from rural populations, and the results of the study should be used with caution when generalizing to urban populations.

## Conclusion

In this registry-based cross-sectional study, we found a structural postponement in marriage age, MCI, and pregnancy age with demographic and regional heterogenicities among nulliparous couples with planned pregnancies in mainland China during 2013–2019. To mitigate the postponement, inclusive and all-round parenting support should be developed and implemented in mainland China, and policy makers should consider heterogenicities within the population to adapt to the postponement of parenthood.

### Electronic supplementary material

Below is the link to the electronic supplementary material.

**Supplementary Material 1:** Supplementary Materials: Definitions of first-tier, new first-tier, second-tier, third-tier, fourth-tier, and fifth-tier cities in mainland China. **Table S1:** Sensitivity analyses in participants without history of pregnancy

## Data Availability

The datasets generated and analyzed during the current study are not publicly available due information governance restrictions in place to protect individuals’ confidentiality but are available from the corresponding author on reasonable request.

## List of abbreviations

NFPCP: National Free Pre-Pregnancy Check-ups Project
LMP: Last menstrual period
MCI: The difference in marriage time and LMP in years
BMI: Body Mass Index
APC: Annual percentage change
AC: Annual changes
GDP: Gross domestic product
