# Supplementary material for: Structural transition of parenthood among Chinese nulliparous couples with planned pregnancies, 2013–2019
Source: BMC Public Health. 2023 Dec 4;23:2412. doi: 10.1186/s12889-023-17380-2 (PMC10696718; doi:10.1186/s12889-023-17380-2)
Supplement: Supplementary file 1 — Supplementary Material 1: Supplementary Materials: Definitions of first-tier, new first-tier, second-tier, third-tier, fourth-tier, and fifth-tier cities in mainland China. Table S1: Sensitivity analyses in participants without history of pregnancy [file 12889_2023_17380_MOESM1_ESM.pdf]

Supplementary Materials

According to the 2020 ranking of cities for commercial attractiveness, cities in mainland China were classified as:

- **First-tier cities:** Beijing, Shanghai, Guangzhou, and Shenzhen;
- **New first-tier cities:** Chengdu, Chongqing, Hangzhou, Wuhan, Xi'an, Tianjin, Suzhou, Nanjing, Zhengzhou, Changsha, Dongguan, Shenyang, Qingdao, Hefei, and Foshan;
- **Second-tier cities:** Ningbo, Kunming, Fuzhou, Wuxi, Xiamen, Jinan, Dalian, Harbin, Wenzhou, Shijiazhuang, Quanzhou, Nanning, Changchun, Nanchang, Guiyang, Jinhua, Changzhou, Huizhou, Jiaxing, jiaxing, Nantong, Xuzhou, Taiyuan, Zhuhai, Zhongshan, Baoding, Lanzhou, Taizhou, Shaoxing, Yantai, and Langfang.
- **Third-tier cities:** Weifang, Yangzhou, Haikou, Shantou, Luoyang, Urumchi, Linyi, Tangshan, Zhenjiang, Yancheng, Huzhou, Ganzhou, Taizhou, Jining, Hohhot, Xianyang, Zhangzhou, Jieyang, Jiangmen, Guilin, Handan, Wuhu, Sanya, Fuyang, Huai'an, Zunyi, Yinchuan, Hengyang, Shangrao, Liuzhou, Zibo, Putian, Mianyang, Zhanjiang, Shangqiu, Yichang, Cangzhou, Lianyungang, Nanyang, Jiujiang, Xinxiang, Xinyang, Xiangyang, Yueyang, Bengbu, Zhumadian, Chuzhou, Weihai, Suqian, Zhuzhou, Ningde, Zhoukou, Ma'anshan, Yichun, Huanggang, Xingtai, Chaozhou, Qinhuangdao, Zhaoqing, Jingzhou, Qingyuan, Tai'an, Suzhou, Anshan, Anqing, Heze, Nanchong, Lu'an, Daqing, and Zhoushan.
- **Fourth-tier cities:** Changde, Weinan, Xiaogan, Lishui, Yuncheng, Dezhou, Xuchang, Xiangtan, Jinzhong, Anyang, Sanming, Kaifeng, Chenzhou, Maoming, Shaoyang, Deyang, Longyan, Nanping, Huainan, Huangshi, Yingkou, Haozhou, Rizhao, Xining, Quzhou, Dongying, Jilin, Shaoguang, Zaozhuang, Baotou, Huaihua, Xuancheng, Linfen, Liaocheng, Meizhou, Panjin, Jinzhou, Yulin, Beihai, Baoji, Wuzhou, Jindezhen, Yulin, Shiyan, Shanwei, Xianing, Yibin, Jiaozuo Pingdingshan, Binzhou, Ji'an, Yongzhou, Yiyang, Qiannan, Dandong, Qujin, Leshan, Qiandongnan, Zhangjiakou, Huangshan, Erdos, Yangjiang, Luzhou, Enshi, Hengshui, Tongling, Chengde, TsitsiharHehe, Yan'an, Wushun, Lasa, Tongren, Changzhi, Dazhou, Erzhou, Yizhou, Lvliang, Huaibei, Puyang, Meishan, Chizhou, Jinmen;
- **Fifth-tier cities:** Hanzhong, Liaoyang, Wuzhou, Ingtan, Baise, Bijie, Qinzhou,Yunfu, Jiamusi, Chaoyang, Guigang, Lijiang, Siping, Neijiang, Liupanshui, Anshun, Sanmenxia, Chifeng, Xinyu, Mudanjiang, Qinzhou, Jincheng, Zigong, Benxi, Fangchenggang, Tieling, Suizhou, Guang'an, Guangyuan, Tianshui, Suining, Pingxiang, Xishuangbanna, Suihua, Hebi, Songyuan, Fuxin, Jiuquan, Zhangjiajie, Qianxi'nan, Baoshan, Xiangxi, Zhaotong, Karamay, Hulun Buir, Hezhou, Tonghua, Yangquan, Hechi, Laibin,

Yuxi, Ankang, Tongliao, Dehong, Chuxiong, Shuozhou, Yili, Wenshan, Jiayuguan, Liangshan, Ziyang, Xilingol, Ya’an, Puer, Chongzuo, Qingyang, Baiyin, Longnan, Zhangye, Shangluo, Heihe, Hami, Wuzhong, Panzhihua, Bayannur, Ulanqab, Baishan, Changji, Baicheng, Xingan, Dingxi, Kashi, Bazhong, Jixi, Wuhai, Lincang, Haidong, Shuangyashan, Aksu, Shizuishan, Alashan, Haixi, Pingliang, Liaoyuan, Linxia, Tongchuan, Jinchang, Hegang, Yichun, Linzhi, Guyuan, Wuwei, Danzhou, Turpan, Ganzi, Zhongwei, Nujiang, Hetian, Diqing, Gannan, Aba, Daxinganling, Qitaihe, Shannan, Rikaze, Tacheng, Bortala, Changdou, Altay, Yushu, Hainan, Kizilsu, Ali, Haibei, Huangnan, Guoluo, Naqu, and Sansha.

Table S1. Sensitivity analyses in participants without history of pregnancy.

Year	Mean (95% CI)	Prevalence in % (95% CI)
**Marriage**		
Total	25.22 (25.22-25.22)	1.18 (1.17-1.18)
2013	24.70 (24.70-24.71)	1.06 (1.04-1.07)
2014	24.87 (24.86-24.87)	1.05 (1.04-1.07)
2015	25.13 (25.12-25.13)	1.07 (1.05-1.08)
2016	25.44 (25.43-25.44)	1.25 (1.23-1.27)
2017	25.63 (25.62-25.63)	1.30 (1.28-1.32)
2018	25.91 (25.90-25.92)	1.43 (1.41-1.46)
2019	26.15 (26.14-26.16)	1.54 (1.51-1.58)
P_{trend}	<0.001	<0.001
AC	0.25 (0.24-0.26)	-
APC	-	7.20 (5.45-8.99)
**Marriage and Conception Interval**		
Total	0.86 (0.86-0.86)	22.61 (22.58-22.64)
2013	0.80 (0.79-0.80)	20.02 (19.96-20.08)
2014	0.77 (0.77-0.77)	19.12 (19.06-19.18)
2015	0.79 (0.79-0.80)	20.39 (20.32-20.45)
2016	0.95 (0.95-0.95)	23.27 (23.19-23.34)
2017	0.99 (0.98-0.99)	26.72 (26.63-26.81)
2018	0.98 (0.98-0.99)	29.02 (28.92-29.12)
2019	1.00 (1.00-1.01)	30.81 (30.68-30.94)
P_{trend}	<0.001	<0.001
AC	0.04 (0.02-0.06)	-
APC	-	8.94 (6.66-11.26)
**Pregnancy Age**		
Total	25.94 (25.94-25.95)	1.95 (1.95-1.96)
2013	25.36 (25.35-25.36)	1.66 (1.65-1.68)
2014	25.52 (25.52-25.53)	1.67 (1.65-1.68)
2015	25.80 (25.79-25.80)	1.68 (1.67-1.70)
2016	26.22 (26.21-26.22)	2.25 (2.22-2.27)
2017	26.46 (26.45-26.46)	2.37 (2.34-2.39)
2018	26.72 (26.71-26.72)	2.45 (2.42-2.48)
2019	27.06 (27.05-27.06)	2.52 (2.48-2.56)
P_{trend}	<0.001	<0.001
AC	0.29 (0.27-0.31)	-
APC	-	8.78 (5.69-11.96)

Note: AC=Annual Change; APC=Annual Percent Change. A total of 12 204 436 participants is included in the sensitivity analyses.
